# An Interplay between Transcription Factors and Recombinant Protein Synthesis in *Yarrowia lipolytica* at Transcriptional and Functional Levels—The Global View

**DOI:** 10.3390/ijms25179450

**Published:** 2024-08-30

**Authors:** Maria Gorczyca, Paulina Korpys-Woźniak, Ewelina Celińska

**Affiliations:** Department of Biotechnology and Food Microbiology, Poznan University of Life Sciences, Wojska Polskiego 48, 60-637 Poznan, Poland; maria.gorczyca@up.poznan.pl (M.G.); paulina.korpys@up.poznan.pl (P.K.-W.)

**Keywords:** heterologous protein, yeast, protein production, omics data, transcriptional regulation

## Abstract

Transcriptional regulatory networks (TRNs) associated with recombinant protein (rProt) synthesis in *Yarrowia lipolytica* are still under-described. Yet, it is foreseen that skillful manipulation with TRNs would enable global fine-tuning of the host strain’s metabolism towards a high-level-producing phenotype. Our previous studies investigated the transcriptomes of *Y. lipolytica* strains overproducing biochemically different rProts and the functional impact of transcription factors (TFs) overexpression (OE) on rProt synthesis capacity in this species. Hence, much knowledge has been accumulated and deposited in public repositories. In this study, we combined both biological datasets and enriched them with further experimental data to investigate an interplay between TFs and rProts synthesis in *Y. lipolytica* at transcriptional and functional levels. Technically, the RNAseq datasets were extracted and re-analyzed for the TFs’ expression profiles. Of the 140 TFs in *Y. lipolytica*, 87 TF-encoding genes were significantly deregulated in at least one of the strains. The expression profiles were juxtaposed against the rProt amounts from 125 strains co-overexpressing TF and rProt. In addition, several strains bearing knock-outs (KOs) in the TF loci were analyzed to get more insight into their actual involvement in rProt synthesis. Different profiles of the TFs’ transcriptional deregulation and the impact of their OE or KO on rProts synthesis were observed, and new engineering targets were pointed.

## 1. Introduction

Transcriptional regulatory networks (TRNs) can be defined as regulatory interactions among transcription factors (TFs) and their target genes [1,2]. A given TRN is responsive to a given stimulus and governs a specific biological process. Skillful manipulation with TRN will presumably lead to global optimization of a microbial cell towards a researcher-defined target. TRNs have not yet been described for *Y. lipolytica*; however, a very insightful metabolic model has been recently released [3], though not yet tested for recombinant protein (rProt) production prediction. In our studies, we are interested in defining TFs, and in further perspective, TRNs, that are involved in enhancing *Yarrowia lipolytica*’s capacity towards high-level production of rProts. The first step in the endeavor is identifying TFs implicated in the process. *Y. lipolytica* has long been used as a microbial platform for rProts production [4,5,6,7]. Thanks to recent achievements in developing an erythritol-inducible expression system [8,9,10,11,12,13], *Y. lipolytica* has become a potent alternative to conventional expression platforms in industrial applications (several companies are implementing this system into their portfolio).

Previously, we have shown that by applying the knowledge-driven genetic engineering of *Y. lipolytica*’s synthesis and secretion mechanisms, the rProt yields can be significantly enhanced [14,15,16]. By defining the genes deregulated upon production of biochemically-different rProt [16] and using them as engineering ‘helper genes’ [15], the target rProt amounts were increased by over 2-fold. Notably, the previous set of the ‘helper genes’ covered those involved in the translation, folding, and trafficking of polypeptides, as well as one TF, Hac1 (*YALI0B12716*; hereafter, gene signatures will be abbreviated by skipping the ‘*YALI0*’ prefix), which is known to initiate the ER-based unfolded protein response (UPR). Hac1 mediates the activation of hundreds of molecular events, including the increased provision of chaperones and membranes, to concertedly relieve the burdened secretory pathway, e.g., [17,18]. Hence, the Hac1-governed TRN is directly associated with the biological process ‘protein synthesis and secretion’ and matches our practical aim of enhancing rProts synthesis. Technically, co-overexpression (co-OE) of *HAC1* with a reporter protein initially led to mediocre yield improvement (of ~30%; [15]), but more in-depth studies showed that the effect was indeed bifurcated, causing a nearly 7-fold drop in the retained fraction of rProt but promoting its secretion by nearly 2.5-fold [19].

The following studies on exploiting *Y. lipolytica*’s TRNs for researcher-defined practical aims were focused on enhancing lipid accumulation [20,21], stress resistance, and rProts synthesis [22,23]. In their high-throughput screens, [20] i.a., identified *Brg1* (*E31757*) as a TF promoting total lipid accumulation by ~1.9-fold when OE (the highest increase read from amongst all TFs tested). Gorczyca et al. [22] proved that the increased abundance of Skn1 (*D14520*) enables maintaining the rProt synthesis capacity under heavy osmotic stress, while OE of the Yap-like TF-encoding gene (*D07744*) positively impacts the growth of *Y. lipolytica* under alkaline conditions. OE of *GZF1* (*D20482*) and *HSF1* (*E13948*) brings universal improvement in the rProts synthesis, irrespectively from the environmental conditions. Deletion (knock-out; KO) of *GZF1* and *HSF1* promotes the synthesis of neutral lipids under a low C/N ratio (C/N of 4; not promoting lipid accumulation). Finally, a comprehensive investigation of a library of *Y. lipolytica* strains over-expressing (OE-ing) one of 125 TFs under 72 different environmental conditions revealed multiple interesting phenotypes driven by the increased abundance of the TFs [23], data presented in a searchable database YaliFunTome (https://sparrow.up.poznan.pl/tsdatabase/, accessed throughout June 2024). *Inter alia*, the YaliFunTome database presents the phenotype of another global enhancer of rProt synthesis, Klf1 (*D05041*), as well as TFs acting against this biological process, Azf1 (*A16841*), Dep1 (*F05896*), and Cat8 (*C19151*), all of which are known to be involved in carbon metabolism [24,25,26,27,28]. Notably, several of the TFs studied there promoted growth under limited oxygen availability (*LAC9* (*D20460*), *B14443*, *B20944*, and *DAL81* (*D02783*)), which is the major limiting factor in *Y. lipolytica* growth and rProt synthesis [29,30,31]. Altogether, those studies proved the validity of the concept of using TFs as tools in driving desired phenotypes in *Y. lipolytica*.

With the current advent of next-generation sequencing technologies, DNA synthesis, and even miniaturization and parallelization of strain culturing and sample analysis, the repositories of ‘big biological data’ have grown enormously. This is specifically true for the model microorganisms, like *Saccharomyces cerevisiae* or *Escherichia coli*. Considering the extent of the datasets deposited in publicly accessible databases, ‘big biological data recycling’ has become a very efficient research approach in driving novel hypotheses and findings, also in the field of yeast research [32]. If approached carefully and with adequate methodology [33,34,35,36,37], reusing previous data enables substantial time- and resource-savings. Examples of successful completion of such analyses for yeast are available [3,38,39]. When compared to the model yeast species, the high-throughput datasets for *Y. lipolytica* deposited publicly are still limited. Nevertheless, some transcriptomics data recycling for *Y. lipolytica* has been very recently reported [11,19,39]; where RNAseq datasets were extracted, arranged in different combinations, and re-analyzed to answer new research questions. Those previous studies reported recycling of transcriptomics data only.

In this study, we aimed to combine and reanalyze datasets related to the expression profile of TFs and the functional effect of the TFs OE on rProt synthesis in *Y. lipolytica*. The previously generated datasets (transcription level of TFs and amounts of rProts triggered by TF’s OE) were juxtaposed to investigate an interplay between TRNs and rProts synthesis in *Y. lipolytica* at transcriptional and functional levels. Having transcriptomics data on one hand and functional screening results on the other, we wanted to test a working hypothesis of whether transcriptomics data could be used as a selection-driver of TF involved in a specific biological process, here—rProt synthesis in *Y. lipolytica*, and if a preliminary prognostic of its mode of implication can be inferred. For several TFs, further experimental verification followed the computational analyses.

## 2. Results and Discussion

### 2.1. Data Extraction, Juxtaposition, and Global Clustering—Clusters Overview

Transcriptomics data were extracted from the NCBI SRA (Sequence Read Archive) database (PRJNA701856 and PRJNA869113). The data represent the relative expression level of a given TF-encoding gene in a *Y. lipolytica* strain OE-ing one of the biochemically different rProt: (i) scSoA—highly disulfide-bonded secreted alpha-amylase from *Sitophilus oryzae*; (ii) scYFP—minimally modified post-translationally secreted small yellow fluorescence protein; (iii) inYFP—the same protein (YFP) in intracellular form; (iv) scTlG—highly glycosylated secreted glucoamylase from *Thermomyces lanuginosus*; or a strain co-OE-ing scYFP and a TF Hac1 [16,19]. The strains were maintained in steady-state, as indicated previously [14,16]. The two strains, scSoA and scYFP, were characterized by high-level synthesis and secretion of the rProts and hence were assigned names and abbreviated as high-level synthesis and secretion, HSS. The other three, inYFP, TlG, and scYFP-HAC1, produced limited amounts of the rProts, and based on transcriptomics data were shown to be facing UPR, hence they were assigned and abbreviated as UPR strains. The raw data were filtered for genes encoding TFs, according to a previously defined list of putative TFs [20]. Only these records were considered further in this study. Differential expression data, used hereafter, express fold change (FC) values calculated as a ratio of the normalized transcript counts (log2) in the OE-ing strain to a prototrophic control, maintained likewise.

Functional data on the amounts of rProt (intracellular fluorescent protein) synthesized by *Y. lipolytica* strains OE-ing a specific TF were extracted from the YaliFunTome database [23]. The data were read as fluorescence of the strain, expressed as total amounts of rProt or normalized per biomass. Differential phenotype data, used hereafter, denote FC values calculated as a ratio of the total or normalized fluorescence readouts in the OE-ing strain to a prototrophic control, maintained likewise.

All the TFs considered in this analysis, along with assigned or putative functions, are given in Table 1. Both datasets generated previously were expanded due to different sets of TFs scoring significant results in the transcriptomics (87) and functional screens (significant data and tendencies—124). Hence, here analyzed dataset is original in terms of extent and also considering the combination of functional and transcriptomics data.

**Figure 1:**
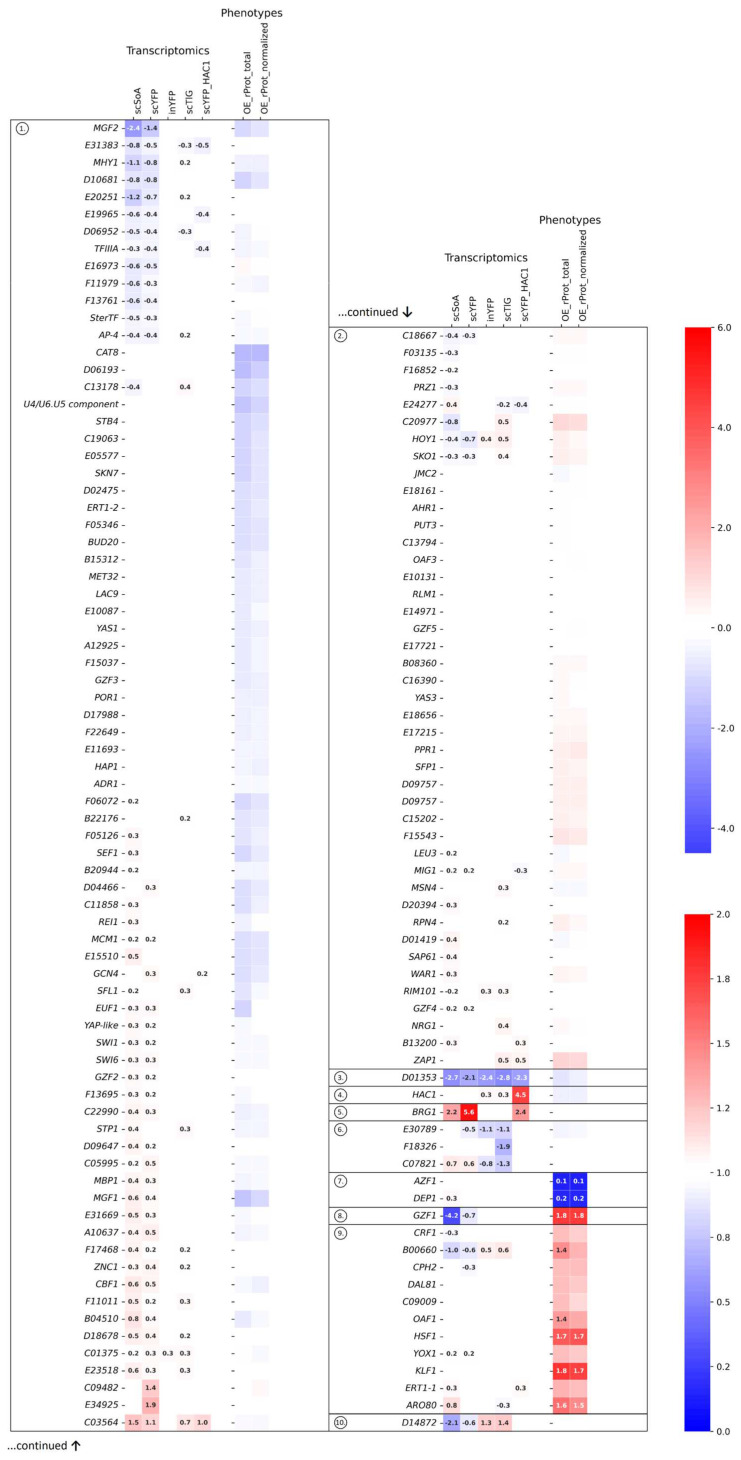
Clustered heatmap of transcriptomics (five sets ‘transcriptomics’) and functional (two sets ‘phenotypes’) data for the 140 TFs analyzed. Each row represents a single TF-encoding gene. Data are color-coded according to the legends on the right. Top legend—transcriptomics (‘0’ and white denotes no change); bottom legend—phenotype (‘1’ and white denotes no change). Numbers in the cells denote the fold change value over the control strain. The lack of numbers indicates a lack of statistical significance. Ten clusters were delimited. In the figure, subsequent clusters are separated by a bold horizontal line. The heatmap was split between clusters 1 and 2 for clarity of presentation.

Based on the overall performance profile comprising both the transcriptomics and functional data, the set of TFs was clustered into 10 clusters (Figure 1; the optimal number of clusters was defined using an elbow method; Figure S1).

As can be read from Figure 1, cluster 1 is the most abundant and contains the most variable set of TFs. It comprises 76 genes exhibiting moderate up-/down-regulation (especially in the HSS strains) or an unchanged transcriptomic profile upon co-OE of any rProt. Their OE leads to an insignificant but generally negative impact on the rProt production phenotype. This cluster comprises several TFs whose function is well-described, and their role in rProt synthesis has been confirmed. For example, Skn7 (*D14520*) was proved to play a role in maintaining the rProt synthesis capacity under osmotic stress infliction [22]. Here, no transcriptional or functional effects were observed as the conditions were not adequate (no osmotic stress simulation). Cluster 1 is enriched in significantly deregulated TFs of known roles in the dimorphic transition, like *MGF2*/*B19602*, *MHY1*/*B21582*, *MGF1*/*D01573*, and *ZNC1*/*B05038* [47,53,64,65]. Their OE concertedly contributed to a slight decrease in the rProt synthesis, though they displayed an opposite deregulation pattern (two former were downregulated and the two latter upregulated in the HSS strains). Two representatives of the Gzf-family (genuine GATA—binding zinc finger family) known to be involved in nitrogen catabolite repression (NCR) were assigned to this cluster as well (*GZF2*/*F17886*—essential for growth on simple nitrogen sources, inducer of NCR; and *GZF3*/*C22682*—known repressor of NCR; [42]). But the other members of the Gzf family were clustered in either cluster 2 (*GZF4*/*E05555*—strongly upregulated under inorganic nitrogen [42], hence—putative inducer of NCR; and *GZF5*/*E16577—*function not investigated) or a self-contained cluster 8 (GZF1/D20482—known inducer of NCR; discussed hereafter). *GZF2* and *GZF4,* although clustered separately, displayed a similar pattern of deregulation (upregulation in HSS strains); likewise, the *GZF3* and *GZF5* genes; however, minor changes to the OE effect contributed to their separation.

**Figure 2:**
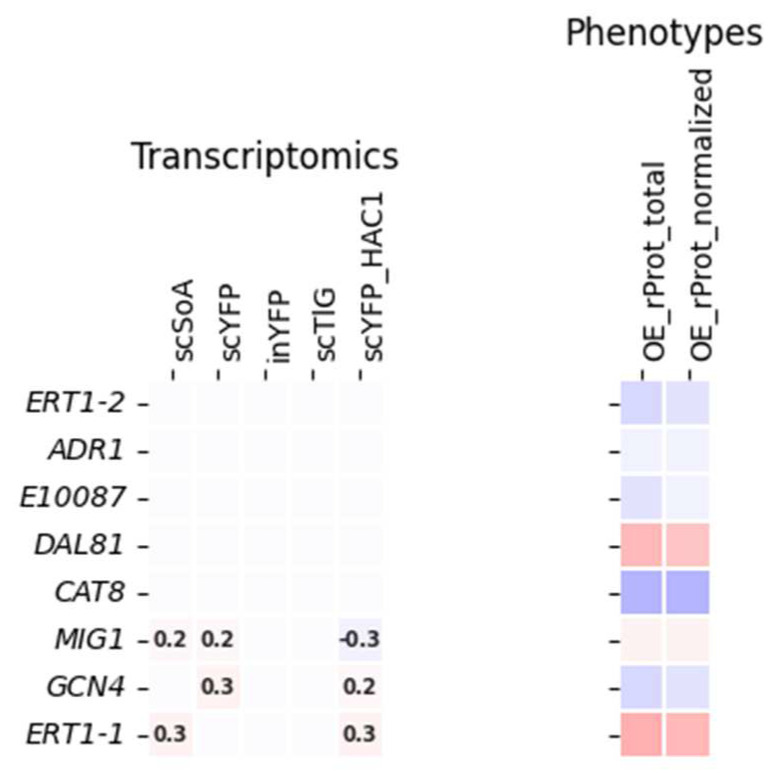
A subgroup of TFs assigned to a ‘cellular response to nutrient levels’ category. Data color-coding and mode of presentation are according to Figure 1, and a legend is presented there.

Of note, several other global regulators of carbon/nitrogen metabolism were assigned to cluster 1, i.e., *ERT1-2*/*E03410*, *GCN4*/*E27742*, *ADR1*/*F21923*, *CAT8*/*C19151*, and *E10087*. Strikingly, none of these genes displayed any specific deregulation pattern in the analyzed transcriptomics data, but their OE contributed universally to the inhibition of rProt synthesis (Figure 1 and Figure 2). Furthermore, several of these genes were assigned to a separate functional category upon running a statistical overrepresentation of GO terms test. Their specific function and involvement in rProt synthesis are discussed in Section 2.2. Cluster 1 also contained 4 genes (*HAP1*/*F17424*, *SKN7*/*D14520*, *SFL1*/*D04785*, *EUF1*/*F01562*) that were either slightly upregulated in the HSS strains or showed insignificant changes in transcriptomic data. OE of these 4 genes uniformly led to a slight, insignificant decrease in the rProt synthesis capacity. Due to their previously documented importance to *Y. lipolytica* phenotype modulation [22,23], they will be subjected to further functional studies (Section 2.4).

Delimited cluster 2 is the second largest cluster, containing 43 TF-encoding genes with low/lack of transcriptional deregulation and a functional phenotype similar to the control strain but with a tendency towards an increase in rProt synthesis upon OE. That cluster contains the two previously mentioned members of the Gzf family involved in the nitrogen metabolism regulation, as well as TFs of known involvement in stress response, like *SKO1*/*C16863*, *YAS3*/*C14784*, *MSN4*/*C13750*, *RIM101*/*B13640*, or a recently described repressor of erythritol utilization, *NRG1*/*C12364* [50]. Consistently, all the ‘stress response’ TFs were upregulated transcriptionally in at least one UPR strain, which was shown to face severe stress. Another interesting gene from this cluster is *ZAP1*/*D23749* (Zinc-responsive transcriptional regulator 1), involved in zinc ion homeostasis by zinc-responsive transcriptional regulation. It displayed upregulation in the UPR strains, and its OE led to an increase in the rProt synthesis (FC 1.19), which makes it a candidate for a ‘helper gene’ worth further functional studies.

A single-gene cluster 3 is represented by the *D01353* gene, which has a unique expression profile. The gene was strongly downregulated upon any rProt OE. Contrary to expectations, its OE had no significant impact on the rProt phenotype. Another single-gene-contained cluster, cluster 4, containing the *HAC1*/*B12716* gene, was delimited mostly because of its strong upregulation in the scYFP-HAC1 strain, induced synthetically [19]. Due to the peculiar, unexpected outcome (lack of induction of rProts synthesis upon OE), this TF was subjected to further functional studies by investigating the Δ*hac1* phenotype (Section 2.4). Cluster 5 also contains only 1 gene (*BRG1*/*E31757*) displaying strong upregulation in HSS and scYFP-HAC1 strains, with no significant change in OE phenotype data. Knowing its role in the synthesis of lipids in *Y. lipolytica* [20], it is tempting to state that its expression was enhanced in the HSS strains in response to high demands for membranes intensively exploited in the vesicular transportation of rProts. Representatives of cluster 6 (*E30789*, *F18326*, and *C07821*) showed strong downregulation in the UPR strains but no change in the OE phenotype. TFs assigned to cluster 7, *DEP1*/*F05896* and *AZF1*/*A16841*, were characterized by minimal changes in transcriptional profile, but both contributed to a distinctive, very strong limitation of rProt synthesis upon their OE. These two were also selected as the candidates for studying Δ*azf1* and Δ*dep1* phenotypes with high expectations (Section 2.4).

Cluster 8 contained only 1 gene (*GZF1*/*D20482*), which exhibits a unique performance, namely, strong downregulation in HSS strains and high over-production of rProt when OE. Gzf1 is a member of the discussed above Gzf-family, acting as an activator of NCR genes. It was previously identified as one of the universal enhancers of rProt synthesis [22]. Considering its putative role as an NCR activator, such an effect could be attributed to the enhanced nitrogen scavenging capacity required for the high-level production of rProt. But then its significant downregulation in the HSS strains is not clear, as those strains definitely encountered nitrogen limitation due to their extensive consumption for rProt synthesis. A previous report detailing the impact of Gzf family members on growth and lipid synthesis by *Y. lipolytica* cultivated in the presence of different nitrogen sources sheds some light on the structure of the Gzf TRN [42]. Primarily, they found that expression of *GZF1*, *GZF2*, *GZF4*, and *GZF5* was increased when the cells were grown on ammonium as the sole nitrogen source, while the level of expression of *GZF3* was not significantly affected. Translating into our data, their expression should be upregulated in the HSS, facing nitrogen depletion. It was true only for the *GZF2* and *GZF4* genes, while *GZF5* escaped this mechanism. The highest amplitude of the upregulation due to nitrogen source type was previously noted for the *GZF1*. In our transcriptomics, *GZF1* displayed a tremendous drop in expression in the HSS strains, which is in contrast to our expectations. Interestingly, [42] showed that when *GZF1* was deleted (Δ*gzf1*), no growth defect on any of the nitrogen sources tested was displayed, suggesting that its function was taken over by the other gene. This could be performed by either *GZF2* or *GZF4,* whose expression was significantly upregulated in the HSS strains. It was also discovered that Δgzf3 (repressor of NCR) led to the loss of regulatability of *GZF1* by the nitrogen source, i.e., *GZF1* expression was elevated irrespectively of the nitrogen provided. The opposite was observed in the Δ*gzf2* background. In our data, *GZF3* was not deregulated under any conditions, which is consistent with the findings by [42]. Though functional *GZF3* and *GZF2* were present in the cell. Hence, *GZF1*, which is of key interest to us as the potent inducer of rProt synthesis, could be regulated by the nitrogen availability. Considering increased demands for nitrogen and the upregulation of *GZF2*, we expected upregulation of *GZF1* expression in HSS strains. The question of why *GZF1* was downregulated in the HSS strains hence remains to be answered. The effect of OE and Δ*gzf1* genotypes on rProt synthesis in *Y. lipolytica* in a direct comparative experiment was investigated and is presented hereafter (Section 2.4).

Representatives of cluster 9 in general exhibited no significant deregulation in transcriptomic data, but their OE led to strong overproduction of rProt. This group of TFs is definitely of the highest practical interest, gathering candidate enhancers of rProts synthesis. Indeed, 2 genes, *KLF1*/*D05041* and *HSF1*/*E13948*, were previously reported as global enhancers of rProt synthesis [22,23]. Interestingly, a similar level of rProt synthesis enhancement was observed for TF *ARO80*/*C18645*, involved in the catabolism of aromatic amino acids [66]. Likewise, *DAL81*/*D02783,* known for its role in nitrogen turnover, was expected to bring a positive effect to rProt synthesis. It is thus concluded that the positive impact triggered by the overrepresentation of Aro80 and Dal81 is associated with the increased supply of the rProt building blocks. By similarity to *KLF1*’s function, the beneficial effect of *CRF1*/*B08206* OE may be attributed to its involvement in oxidative stress response.

An interesting expression profile was observed for the gene *D14872* assigned to a self-contained cluster 10. The gene showed a strong and inverted deregulation trend in the HSS (downregulated) and UPR (upregulated) strains, however, yielding no significant impact on phenotype upon OE. Genes displaying inverted deregulation patterns were deemed to play a direct role in the analyzed biological process (rProt synthesis), which turned out to be not the case, at least for *D14872*. They will be combined and analyzed separately in Section 2.3.

### 2.2. Statistical Overrepresentation Test Delimited Two Major Categories amongst TFs

The total set of 140 TFs was subjected to a statistical overrepresentation of biological processes test [67,68]. Expectedly the majority of output records were assigned to ‘regulation of DNA-templated transcription’ or ‘regulation of RNA biosynthetic process’ categories, or related (altogether 31 categories and subcategories comprising a variable number of genes, with a significant enrichment fold from >6 to >17; Table S1).

Interestingly, six genes were assigned to a ‘cellular response to nutrient levels’ category (altogether 8 categories and subcategories comprising a variable number of genes, with a significant enrichment fold from >6 to >13; Table S1). These were transcriptional activators of gluconeogenesis *ERT1-1/2* (cluster 9 *E18304*/ cluster 1, *E03410*), *DAL81* (*D02783*; cluster 9), *GCN4* (*E27742*; cluster 1), a *MIG1*-related regulatory protein (*E07942*; cluster 2), and unknown TF *E10087* (cluster 1; Figure 2). It was the only significantly enriched category, other than related to DNA transcription/RNA biogenesis.

The majority of TFs assigned to that category showed no transcriptional deregulation due to rProt OE. Also, the induced phenotypical changes were rather tendencies. Though the patterns behind them are interesting to track. *ERT1* acts as a positive regulator of gluconeogenesis and fermentable carbon utilization but mechanically is a repressor of transcription by RNA polymerase II by a nonfermentable carbon source. An increased abundance of *ERT1-1* (*E18304*) was recorded in transcriptomes of scSoA and scYFP-HAC1; when overexpressed (OEd), it triggered enhanced rProt synthesis. The other homolog, *ERT1-2* (*E03410*), did not show any changes at the transcriptional level in the analyzed transcriptomes, and its OE led to a slight decrease in rProt amounts. Another gene from this category, *DAL81*/*D02783*, is a positive regulator of genes in multiple nitrogen degradation pathways, involved in nitrogen catabolite activation of transcription from the RNA polymerase II promoter. Even though previously we reported on enhanced protein degradation and deregulation of nitrogen catabolism processes in the UPR strains [16], and enhanced demand for nitrogenous compounds was expected for the HSS strains, its expression level was not changed. However, functional screens revealed its minor but positive effect on rProts synthesis upon OE, which is biologically well-understood. The lack of transcriptional response but a visible effect at the functional level suggests its regulation at the posttranslational level. Another TF from this functional category, *MIG1*/*E07942*, is known to be involved in glucose repression by negative regulation of transcription by RNA polymerase II by glucose. Mig1 (together with Mig2) also induces filamentation under glucose starvation stress. The steady-state maintained cells did not face the main carbon source starvation, as its residual levels were very high. *MIG1* expression was only slightly upregulated in the HSS strains, expected to be in very high demand for carbon building blocks and energy. Its OE led to a minor positive effect on rProts synthesis by *Y. lipolytica*. However, due to its known fundamental role in shaping cellular metabolism, the Δ*mig1* genotype was constructed, and its effect on rProt synthesis was tested (Section 2.4).

All the remaining TFs assigned to the ‘cellular response to nutrient levels’ category (based on enrichment tests or added manually—*CAT8* and *ADR1*) collectively contributed to slightly decreased rProts synthesis upon their OE. Among them, *GCN4* (*E27742*) is a master regulator involved in multiple biological processes, with a primary role in TRN controlling amino acid metabolism. It was also shown to be required for increased biosynthesis of translation precursors such as ribosomal proteins, amino acids, and purines, depending on the external stimuli. Upon amino acid starvation, it plays a key role in the transcriptional induction of almost all genes involved in amino acid biosynthesis (19 per 20 pathways). Notably, it was reported that OE of *GCN4* leads to a reduction in protein synthesis capacity via negative regulation of ribosomal protein gene transcription [48]. Consistently with its assigned role, its OE triggered a slight decrease in the rProt synthesis levels in *Y. lipolytica*. It is known that its levels are strictly regulated at translational/protein and mRNA stability levels (it is constitutively expressed at a low basal level), so the generally observed lack of transcriptional deregulation upon rProts overproduction is not surprising, though the inflicted conditions seem to be relevant stimuli.

TFs *CAT8*/*C19151* and *ADR1*/*F21923* are known to be involved in the regulation of carbon catabolite repression, but in the opposite direction than Mig1. They share the common lack of deregulation upon any rProt OE and the negative effect on rProt synthesis upon OE. Cat8 is an inducer of gluconeogenesis, binding to cis-regulatory elements upon glucose starvation. Adr1 induces transcription of genes involved in alternative carbon utilization upon glucose starvation. Its fundamental role in controlling the Euf1-dependent erythritol utilization cluster in *Y. lipolytica* has been recently reported [11].

Altogether, the above-discussed transcriptional-functional profiles of the genes assigned to a specific functional category ‘cellular response to nutrient levels’ suggest that enhanced degradation of nitrogenous compounds is beneficial for rProts synthesis (Dal81), but enhanced signaling of nitrogen starvation is not (Gcn4). Likewise, the induction of alternative carbon utilization is detrimental.

### 2.3. Usefulness of Specific Deregulation Patterns as Selectors of TFs Involved in Specific Biological Processes

**Figure 3:**
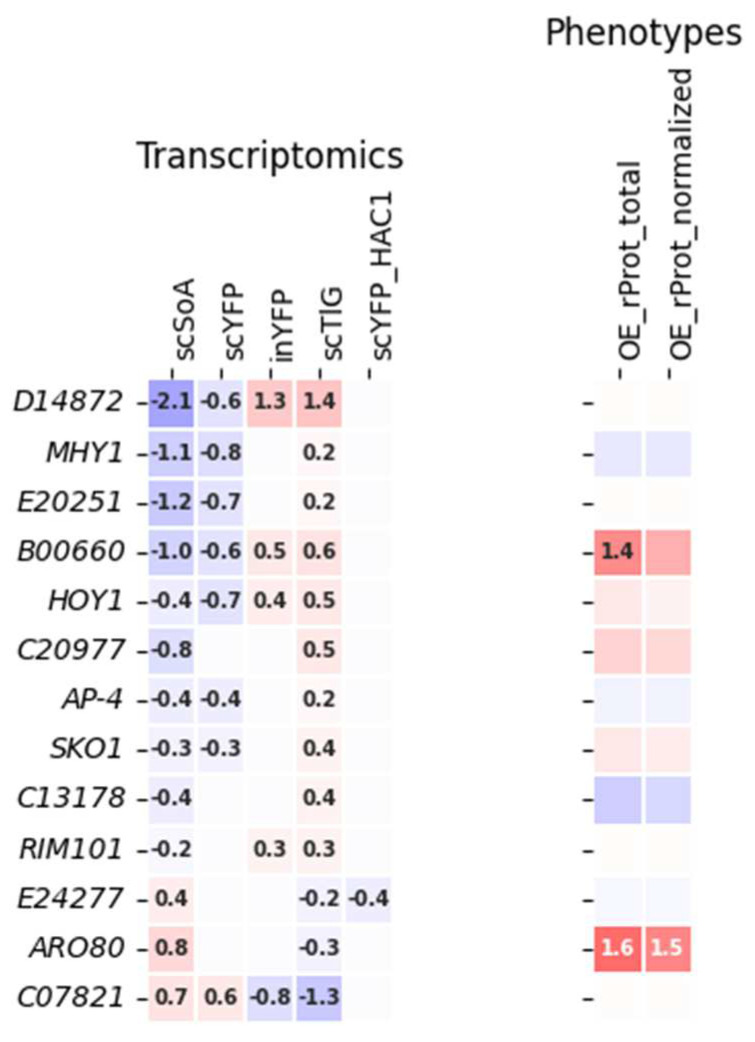
A subgroup of TFs displaying an inverted deregulation pattern in the transcriptomic data of HSS and UPR strains. Data color-coding and mode of presentation are according to Figure 1 and a legend presented there.

**Figure 4:**
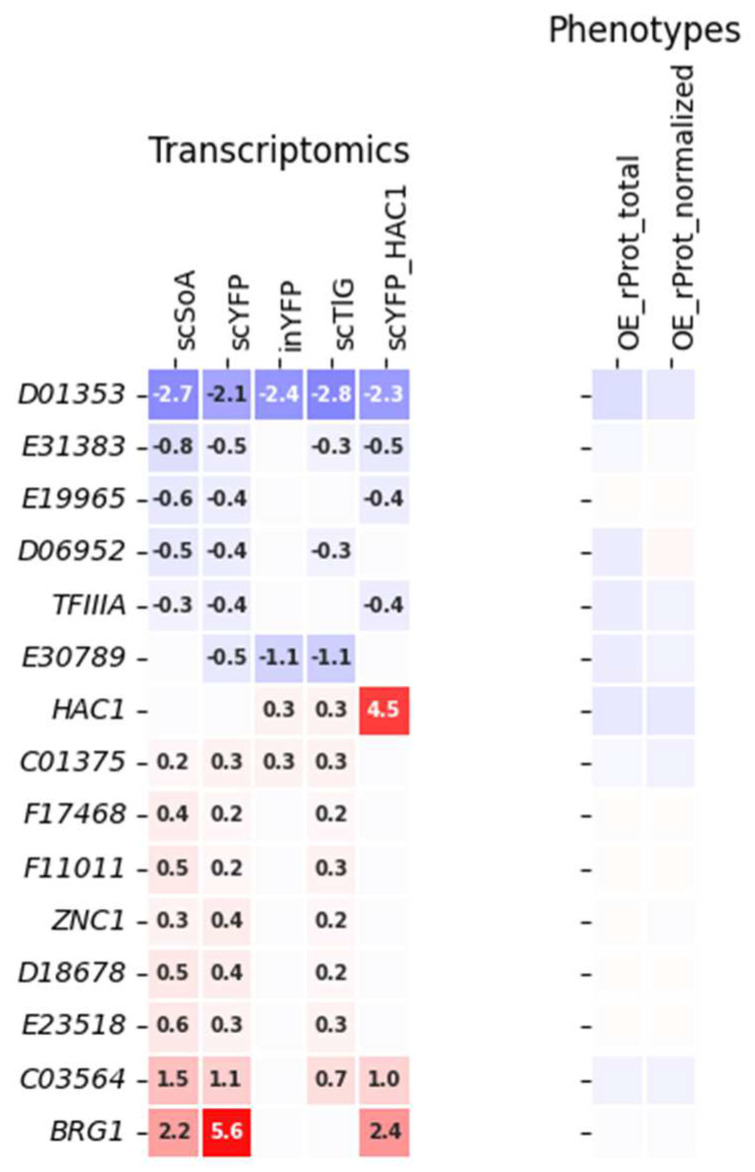
A subgroup of TFs displaying a uniform deregulation pattern in the transcriptomic data (at least 3 out of 5 datasets). Data color-coding and mode of presentation are according to Figure 1, and a legend is presented there. The correlation matrix for these data is shown in Figure S2.

To gain more insight into the relationship between the transcriptional profile and the functional data, the dataset was further filtered to extract and group together TFs that: (i) exhibited inverted transcriptional deregulation patterns in HSS and UPR strains (Figure 3); (ii) showed a uniform deregulation profile, irrespective of the inflicted perturbation (type of rProt) (Figure 4). The phenotypic effect of their OE is shown together with their expression profile in Figure 3 and Figure 4.

The inverted deregulation pattern of the TFs (grouped in Figure 3) suggests that they are directly transcriptionally responsive to the conditions faced and differing the two groups of strains (‘HSS’ and ‘UPR’). Considering the previous results, we presumed that the amounts of rProts and the associated background biological processes (oxidative stress and UPR) were the delimiting factors [16,19], ergo, these TFs are directly involved in these processes. Indeed, *HOY1/A18469, MHY1/B21582*, and *SKO1/C16863* are known for their role in stress response, and these were consistently upregulated in the UPR strains. In this context, both the transcriptional response and functional effect on rProts synthesis upon OE of TF *B00660* are very interesting, considering its putative role in sterol uptake [20]. As evidenced [61], the enhanced sterols residence in the plasma membrane is important for enhanced stress resistance. The here presented dataset suggests that the overrepresentation of TF *B00660* (either natively due to UPR or synthetically by OE) contributes to generally enhanced rProts synthesis, presumably associated with enhanced stress resistance. Unfortunately, many of the remaining genes exhibiting such inverted deregulation patterns are of unknown function, and hence the mechanism behind the phenotypes cannot be deduced.

Generally, two distinct expression profiles could be distinguished for the TFs grouped in Figure 3, those upregulated in the UPR-encountering strains (inYFP, TlG, scYFP-HAC1) and downregulated in HSS (scSoA, scYFP) (majority of the examples), and those upregulated in the former and downregulated in the latter (three TFs: *E24277, C18645*, and *C07821*). If the expression profile could be directly translated into the functional outcomes, then two distinct functional behaviors would be expected. However, that was not the case. For *D14872*, exhibiting significant downregulation in the HSS and significant upregulation in the UPR-facing strains, no functional effect of its OE on rProt synthesis was observed. On the other hand, TFs *B00660* and *HOY1*, while showing the same transcriptional pattern, their OE triggered enhanced rProt synthesis in the host strain (significant or just a tendency). Corresponding enhancement in rProt synthesis was observed for TF *C18645*, whose expression was significantly enhanced in HSS and decreased in the UPR strains. But foremost, the majority of TFs for which the inverted deregulation pattern was observed (so its direct implication in the rProt synthesis was presumed) did not render any significant functional phenotype when OE (only tendencies; Figure 3). Such an outcome implies that our working hypothesis stating that transcriptomics data for the TFs displaying inverted deregulation patterns could be used as a selection-driver of TF involved in a specific biological process, here—rProt synthesis in *Y. lipolytica*, should be partly rejected (for TFs displaying such an expression profile). Furthermore, considering that the TFs exhibiting an inverted deregulation pattern exert similar functional outcomes when OE (*B00660* and *C18645*) implies that the transcriptomics pattern cannot be used as a preliminary prognostic of the TF’s mode of implication (inducer/repressor) in the biological process under study.

Another dataset prepared to test our working hypothesis comprised TFs that were responsive to enhanced synthesis of any rProt, irrespective of the biochemical character of the rProt and the background biological processes awakened (Figure 4). These comprised TFs globally up- or down-regulated across all the transcriptomes tested (in at least 3 out of 5). As can be read from Figure 4, global responsiveness to the over-synthesis of rProt is not an optimal prognostic of direct involvement of a TF in rProt synthesis. Indeed, the majority of TFs, when OE, did not exert any significant effect on rProt synthesis. Nevertheless, some significant correlation between the TFs transcriptomics profile and the rProt amounts upon TF’s OE was noted (scSoA, r = 0.6, inYFP, r = 0.56, scTlG, r = 0.63; significant at *p* < 0.05; Figure S2). It suggests that the TFs that were downregulated in the transcriptomes contribute to a decrease in the rProts synthesis when OEd, and that TFs upregulated promoted rProt synthesis when OEd; so such data could be used as a careful hypothesis driver on the implication of a given TF in the biological process under study (rProt synthesis in this case).

### 2.4. Direct Comparison of Phenotypes Elicited by Y. lipolytica Strains Bearing OE or KO of Selected TFs

**Figure 5:**
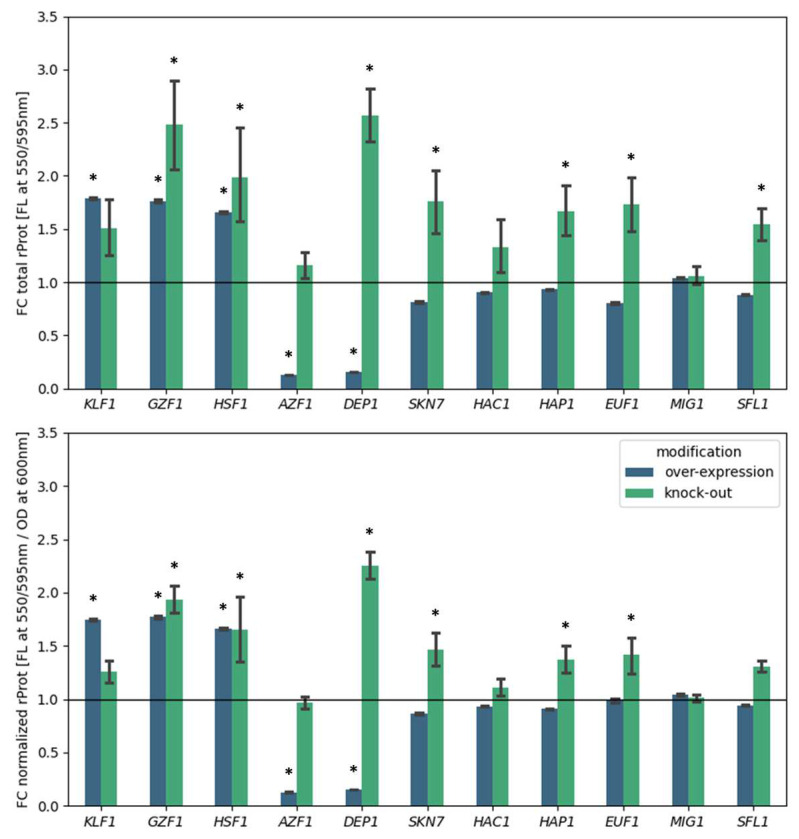
Change in the rProt synthesis capacity investigated in direct comparative functional studies of strains bearing OE (navy) and KO (green) of a given TF. Top chart–fold change (FC) in the total amounts of rProt synthesized by the engineered strains over the control strain, read as fluorescence from the reporter protein. Bottom chart–fold change (FC) in the total amounts of rProt synthesized by the engineered strains over the control strain, read as fluorescence from the reporter protein and normalized per accumulated biomass (read as absorbance at 600 nm). The horizontal line at the level of 1.0 indicates the level of rProt by the control strain. Bars indicate mean values from 2 to 6 subclones cultivated in technical duplicate ± SD. Asterisks (*) indicate a statistically significant difference between the modified strain and the control strain at *p* < 0.05.

In the next step, 11 TFs representing different clusters from Figure 1 were further investigated through direct comparative phenotype reading of *Y. lipolytica* strains bearing their OE and KO, along with rProt OE. Altogether 11 TFs were studied using this approach, selected based on the observed transcriptional-functional profiles (Figure 1) and literature data. These were representatives of the most abundant cluster 1 (*HAP1*/*F17424*, *SKN7*/*D14520*, *SFL1*/*D04785*, *EUF1*/*F01562*), cluster 2 (*MIG1*/*E07942*), cluster 4 (*HAC1*/*B12716*), cluster 7 (*DEP1*/*F05896*, *AZF1*/*A16841*), cluster 8 (*GZF1*/*D20482*), and cluster 9 (*KLF1*/*D05041*, *HSF1*/*E13948*). After genotype construction, selected subclones were cultivated under ‘standard conditions’ (no stress factor inflicted; Section 4.1 and Section 4.2). Results on the total rProt amounts, or normalized per biomass, are shown in Figure 5.

Based on the transcriptional-functional performance patterns (Figure 1), the representatives of cluster 1 (*HAP1*/*F17424*, *SKN7*/*D14520*, *SFL1*/*D04785*, *EUF1*/*F01562*) and cluster 2 (*MIG1*/*E07942*) were subjected to further studies with rather low expectations (no transcriptional deregulation, no effect of the TF’s OE on rProt synthesis). Their selection was based solely on literature data and their important role in shaping the *Y. lipolytica* phenotype [11,22,23,40,49,50,51,57,69,70,71]. Strikingly, the deletion of TFs from cluster 1 led to moderate (55 to 76%) but significant (*p* < 0.05) improvement in the rProt synthesis. Suggesting that the observed tendencies in the functional screens (Figure 1) are indeed valid observations. No significant effect (nor even a tendency) was observed when *MIG1* was either OE or KO (also selected based on literature data and not experimental evidence provided here [57,72,73]). It is presumed that the OE/KO *MIG1* strains should be tested under a spectrum of different nutrient types and levels to observe a valid phenotype.

Hap1 is known to play a fundamental role in managing aerobic metabolism in *S. cerevisiae*. It is responsible for sensing the oxygen levels via the heme signaling pathway, and activation of the oxidative stress response genes [49]. Notably, its OE had a beneficial effect on rProt synthesis in *S. cerevisiae* [74]. It was suggested that the effect was caused by diminishing oxidative stress responses awakened by intensive rProt synthesis. The contradictory effects observed here with *Y. lipolytica* may result from a different metabolism of the two species regarding oxygen demands.

Skn7 is a TF involved in protein secretion and activation of oxidative and osmotic stress responses [75,76]. Previously, its KO in *Y. lipolytica* had a detrimental impact on the strains’ resistance to osmotic stress, irrespective of inflicted anaerobiosis stress, pH, or adopted temperature, leading to a severe underperformance in terms of rProt synthesis once osmotic stress was inflicted [22]. On the other hand, its OE enabled the maintenance of rProt synthesis under high osmolarity and low aeration [22]. Under the current experimental setup (no stress factor, high aeration), the Δ*skn7* phenotype performs better than the control in terms of rProt synthesis. We presume that the adopted conditions contributed to that outcome. A corresponding effect was observed when TF *SFL1*/*D04785* was deleted (Figure 5). Sfl1 (Suppressor Gene for Flocculation) acts as a repressor of filamentous growth and flocculation, antagonizing *FLO* gene action; it is also known to activate the expression of stress-responsive genes [69,70,71]. The beneficial effect of Δ*euf1* is also not yet clear. Euf1 is a master regulator of the ‘erythritol utilization cluster’ [11,50,51] known to govern the expression of several genes involved in erythritol utilization once glucose or glycerol are depleted. Considering the effects observed after KO of TFs from cluster 1, the only explanation for the moment is a slight relief of the transcriptional–translational machinery due to the limited expression of several genes or inadequate conditions enabling a relevant phenotype expression. Collectively, it is tempting to state that once stress conditions are not inflicted, deletion of the global stress-response regulator is beneficial for the costly biological process—rProt synthesis.

The observed Δ*hac1* and *HAC1*-OE phenotypes escaped our expectations, yielding no significant changes. Hac1 mediates the deregulation of hundreds of genes involved in polypeptide formation, folding, and maturation, but also in lipid synthesis, membrane expansion, and many others [17,18,77,78,79]. Hence, along with Hsf1, it is the most frequent target of genetic modifications for enhanced rProt synthesis in yeast [15,19,80,81,82,83,84,85]. As presented in Figure 1, the transcriptional profile of *HAC1* depends on the type of rProt being synthesized due to the background molecular mechanisms. Generally, previously conducted estimations of the rProt amounts synthesized when *HAC1*’s expression was elevated or not showed that the yields of rProts were higher when *HAC1* expression was silenced [14,16]. In the following studies, we attempted to explain this observation. Investigation of Hac1 regulome [19] indicated that elevated Hac1 presence induces massive vacuolar proteolysis. In contrast, in other experiments, we observed a ~30% increase in the rProt synthesis (small, non-modified post-translationally protein, targeted for secretion; scYFP) [15]. The following studies, conducted with cells maintained in the steady state, proved that the effect was indeed bifurcated, causing a nearly 7-fold drop in the retained fraction of rProt but promoting its secretion by nearly 2.5-fold [19]. The protein used in the functional screens (results presented in Figure 1 and Figure 5) has a similar biochemical characteristic to that former but is destined for intracellular retention. We presume that it is the cause of the surprising lack of effect from Δ*hac1* and *HAC1*-OE strains.

Comparable functional outcomes regarding rProt amounts were expected from strains deleted for *AZF1*/*A16841* and *DEP1*/*F05896* (cluster 7), as both the OE strains displayed a significant decrease in rProt synthesis capacity (Figure 1). However, a directly inverted phenotype was observed solely for the *DEP1*-deletant (Δ*dep1*), with no effect from the Δ*azf1* strain (at least in terms of rProt synthesis) (Figure 5). Notably, the Δdep1 rendered the highest increase in total rProt or normalized rProt measures, making this modification the most successful example presented here. Dep1 (Deregulated Expression of Phospholipid biosynthesis) was previously found to enhance the accumulation of lipids in *Y. lipolytica* [20] and specifically—activate phospholipid biosynthesis in *Fusarium* sp. [26]. In contrast, [86] identified Dep1 as a repressor of phospholipid synthesis genes (e.g., *INO1*, *CHO1*, and *OPI3*) in *S. cerevisiae*. It seems that Dep1 from *Y. lipolytica* operates in a similar way as in Fusarium rather than the model yeast species. To our interpretation, an interplay between globally promoted carbon metabolism and enhanced nitrogen scavenging (like in the case of Gzf1/Gzf2+Gzf4 discussed above) may be the molecular mechanism behind the Δ*dep1* phenotype.

For the second representative of cluster 7, *AZF1*/*A16841* (asparagine-rich zinc finger protein), such a directly inverted phenotype regarding rProt synthesis was not observed. Azf1 is a carbohydrate-sensing TF, and in the presence of glucose, it activates genes involved in growth, carbon metabolism, and filamentation in *S. cerevisiae* and *O. polymorpha* [25,28]. In *Y. lipolytica,* the Δ*azf1* mutation rendered no aberrant phenotype in terms of rProt synthesis. This observation is consistent with our previous notions about this phenotype investigated under different cultivation conditions [16,19]. As in the case of Mig1, we presume that alternating carbon sources would enable a relevant phenotype display (but maybe not necessarily related to rProt synthesis).

An unexpected effect was observed upon direct comparison of the strains with either OE or KO of the ‘universal rProt synthesis enhancers’, *KLF1*/*D05041*, *GZF1*/*D20482*, and *HSF1*/*E13948*. Surprisingly, the rProts synthesis capacity was enhanced regardless of whether the genes were OE or KO. Gzf1 was selected for further phenotype studies due to its surprising expression pattern in the HSS strains and highly positive effect on rProt synthesis. Considering the data presented in Figure 1 (decreased expression in the HSS producing a high amount of rProts and enhanced production of rProts when *GZF1* was OE), the data presented in Figure 5 stay in peculiar agreement—if *GZF1* is downregulated (HSS strains), rProts synthesis is high, and when *GZF1* is upregulated (OE)—rProt synthesis is also high. Previous analysis of OE/KO *GZF1* phenotype under various environmental stress factors infliction [22] demonstrated that OE of *GZF1* led to very high and universal enhancement in rProt synthesis. The effect of *GZF1* KO was less uniform—under specific conditions, growth was limited, but some indications for enhanced rProt synthesis capacity were revealed, but only under specific conditions not applied here (low oxygen and hyperosmolality) [22,23]. The primary difference between those previous and current studies relates to cultivation parameters (type of vessel, aeration, buffering system), and foremost—the type of nitrogen source used, which is a relevant factor affecting *GZF1* activity (previously—ammonium sulfate; at present—a 1:1 mixture of glutamic acid and ammonium sulfate). Since that former study, our protocol for *Y. lipolytica* has been greatly improved [31]. We presume that this new cultivation system allows for the actual genotype-driven phenotype development, and the type of nitrogen source used accounts for the observed discrepancies in the Δ*gzf1* phenotype.

Representatives of cluster 9, *KLF1*/*D05041* and *HSF1*/*E13948*, did not display any significant transcriptional deregulation profile in the analyzed transcriptomes, but their OE led to significant enhancement in rProts synthesis capacity (Figure 1). When deleted, Δ*klf1* showed no changes vs. the control strain in terms of rProts production. The same effect was observed previously, using a slightly different cultivation protocol [23]. Klf1 was shown to be responsive to external nitrogen levels in *Schizosaccharomyces pombe*, and its regulome was implicated in cell wall renewal, oxidative stress response, glycolysis, nutrient uptake, RNA-mediated chromatin silencing, glycosidation, and methylation [87]. It was demonstrated to specifically react to oxidative stress via relocalization to the nucleus [88]. That would explain why the rProt synthesis-promoting effect of *KLF1* OE could be seen, even though its expression remained at the control level (Figure 1).

*HSF1*/*E13948,* as the key regulator of global stress response, is the most intensively studied TF in terms of its effect on rProt synthesis in yeast. rProts production was significantly enhanced in *S. cerevisiae* upon OE of *HSF1* in its native or a constitutively active form, *HSF1*-R206S [89,90,91]. Previously, we demonstrated a universal, promoting effect of wild-type *HSF1* OE on rProt synthesis in *Y. lipolytica* [22,23]. Genotype Δ*hsf1* displayed severely impaired growth under both ‘stressful’ and ‘optimal’ growth conditions. The effect was associated with limited production of rProts, but mainly when harsh stress was inflicted. The results here obtained were hence surprising and difficult to explain even with the differences in the growth conditions. Definitely, more studies focused on this aspect must be conducted to get an insight into the molecular mechanisms behind that observation, specifically, whether the other global regulators of stress response were not induced in the Δ*hsf1 Y. lipolytica* strain.

## 3. Summary and Conclusions

Integration of multidimensional, essentially diverse biological data imposes a challenge, as the biological processes are rarely linear. However, with the current accumulation of biological knowledge, such a data-recycling approach is reasonable and, if followed with adequate methodology, may drive novel hypotheses and interesting findings that otherwise could be missed. The approach has become popular with the model organisms, and now, considering how much information is stored in public repositories, it is feasible with nonconventional species like *Y. lipolytica*.

In our recent studies, we investigated the possibility of using TFs as tools for global optimization of rProts production in *Y. lipolytica*. Here, we recycled and integrated datasets related to the expression profile of TFs and the functional effect of their OE on rProt synthesis. Specifically, we were testing a hypothesis of whether transcriptomics data could be used as a selection driver of TF involved in this particular biological process.

The conducted analyses showed that many of the TFs with previously documented implications in rProt synthesis did not respond at the transcriptional level (e.g., Skn7, Hap1). We presume that it was due to conditions under which the transcriptomics samples were collected, which were not activating a given TF’s TRN. This statement is corroborated by the results of further experimental studies. The deletion of TFs from cluster 1 (showing no transcriptional deregulation upon rProt synthesis) led to significant improvement in the rProt synthesis capacity. Likewise, representatives of cluster 7, showing no transcriptional response to rProts synthesis, were shown to strongly decrease this process when OE. Specifically, the deletion of *DEP1* rendered the highest increase in rProt production, demonstrating on one hand its direct implication in this process and on the other—making this modification the most successful example presented here.

The direct correlation between the transcriptional profiles and functional effects of the TF’s OE was either not significant or weak. It was found true only for the TFs showing a uniform pattern of deregulation. In that specific case, TFs that were downregulated in the transcriptomes contributed to a decrease in the rProt synthesis when OEd, and TFs upregulated—promoted rProt synthesis when OEd. Hence, such data could be used as a careful hypothesis driver on the implication of a given TF in the biological process under study (rProt synthesis in this case). In contrast, examples of TFs showing different deregulation patterns but the same functional effect (e.g., TFs of known roles in the dimorphic transition, or *B00660* vs. *C07821* or *C18645*) and the opposite behavior (similar transcriptional pattern but different functional effect; e.g., *HOY1* vs. *MHY1*) were predominant. The intrinsic pleiotropic activity of TFs is the key explanation for this observation (e.g., Gcn4, which is an activator and a repressor).

Nevertheless, the presented data integration led to a new hypothesis and pointed to new directions for experimental studies. For example, the juxtaposition of transcriptional profiles and functional OE results highlighted the putative importance of TF *ZAP1/D23749* for rProt synthesis in *Y. lipolytica*. Likewise, *C18645*, a TF of yet undefined function, was clustered together with the most potent rProt enhancers Hsf1 and Klf1, identified previously. A new research question that remains to be answered through further insightful experimentation is why *GZF1*, an efficient enhancer of rProt synthesis, was indeed transcriptionally downregulated in the strains producing rProts in high quantities. Though it is known to be an activator of NCR. The analysis of transcriptional profiles and functional outcomes of the genes involved in nitrogen metabolism in *Y. lipolytica* implied that the enhanced degradation of nitrogenous compounds is beneficial for rProts synthesis (e.g., Dal81, Aro80), but enhanced signaling of nitrogen starvation is not (Gcn4). Likewise, the induction of alternative carbon utilization is detrimental (e.g., Adr1, Cat8, Ert1-2).

Altogether, this investigation demonstrates the feasibility and potential of biological data recycling and integration for *Y. lipolytica*. It appears that the amount of knowledge accumulated is sufficient, and such an approach leads to novel findings that were not discovered when the datasets were analyzed individually.

## 4. Materials and Methods

### 4.1. Microbial Strains Used for Transcriptomics and Functional OE Screens Data Acquisition

Strains used for transcriptomics and functional OE screen data acquisition were constructed previously [14,16,19,20,23,92]. Briefly, the ‘transcriptomics’ strains are all derivatives of Po1h (Genotype: *MatA, ura3-302, xpr2-322, axp1-2, leu2-270::LEU2*, Phenotype: Ura-, Δ*AEP*, Δ*AXP*, Suc+). The control strain (Po1h_Ura3) was transformed with a solo *URA3* marker cassette to generate a prototroph. Recombinant *Y. lipolytica* strains were transformed with Golden Gate Assembly [93,94] cassettes bearing a single transcription unit each with one target gene: alpha-amylase—SoA, glucoamylase—TlG, fluorescent YFP (intracellular in-/secretory sc-), expressed individually under the control of a synthetic hybrid promoter 4UASpTEF, or co-OEing scYFP and *HAC1* under the control of the pTEF promoter [14,16,19]. Secretory enzymatic reporters were transcriptionally fused with a signal peptide sequence native to exo-1,3-beta-glucanase (*B03564*) [95]. Strains for the ‘phenotype’ screens were constructed by [20,92]. Briefly, a collection of 125 *Y. lipolytica* strains OEing individually one of the TFs and a reporter protein (RedStar2) was created in the background of the JMY2566 (*MATa, ura3::pTEF-RedStar2-LEU2ex-Zeta, leu2-270, xpr2-322*) strain. Both genes were cloned under a constitutive promoter pTEF and were integrated in a zeta platform at the *URA3* locus. Strain JMY2810 (complete prototroph OEing the reporter r-Prot solely; *MATa, ura3::pTEF-RedStar2-LEU2ex-Zeta-URA3ex-pTEF, leu2-270, xpr2-322*) was used as the control for data normalization and calculating FC values. The methodology of the above indicated strain cultivation strategy, sample acquisition, and data analysis was detailed previously [14,16,23,31,96].

### 4.2. Microbial Strains Used for Functional Studies—KO in a Specific TF loci

Microbial strains used for functional studies were partly constructed previously (Gzf1, Hsf1, Skn7, Klf1, Azf1, Dep1) [22,23], and partly were constructed originally in this research (Mig1, Euf1, Sfl1, Hap1, Hac1). All the strains were (re)cultured under the improved cultivation protocol [23,31] to gain a direct comparison of the phenotypes.

#### Construction of the KO Strains—Deletion Cassette and CRISPR-Cas9 Targeting

Standard molecular biology protocols were followed in this study [97]. The deletant strains (KO-TF) were constructed in the background of the JMY2810 strain by disrupting the indicated TF locus. The deletion cassettes were designed using a GoldenGate scaffold indicated in [93] limited to three fragments cloning: (i) ARM up, (ii) NATr (nourseothricin), and (iii) ARM down, flanked with A and B and C and M overhangs. DNA fragments to be cloned were amplified using Phire Hot Start II DNA Polymerase (Thermo Fisher Scientific, Waltham, MA, USA). The cassettes were assembled using a previous protocol for the GoldenGate reaction [93]. White colonies were verified for correctness of the assembly by PCR of adjacent elements and restriction digestion of isolated plasmids. After release from the pSB1A backbone by *Not*I digestion, the cassettes were used for the transformation of *Y. lipolytica* JMY2810.

To increase targeted integration, the CRISPR-Cas9 vector JME4580 [98,99] with the TF-targeting sgRNA oligonucleotide was co-transformed along with the disruption cassette. The methodology followed the previously described protocol [22]. The sgRNA oligonucleotides were designed using the CRISPR design tool from the Benchling platform (https://benchling.com/ (accessed on 31 July 2024Day)). Targeting regions were selected close to the center of the coding sequences. sgRNA oligonucleotides with the highest efficiency scores and lowest number of off-target sites were selected. The 20 bp long target sequences were flanked with *BsmB*I recognition sites and with 4-bp overhangs, enabling their correct integration in a plasmid [99]. Before cloning, the sgRNA oligonucleotides were annealed and then ligated to the JME4580 plasmid using the GoldenGate thermal profile, using *BsmB*I and T4 DNA ligase from New England Biolabs (NEB Ltd., Ipswich, MA, USA). The reaction was then transformed into *E. coli DH5alpha,* and transformants were selected on LB ampicillin agar plates. White clones were screened for correctness by PCR and restriction digestion with *Bgl*II (Thermo Fisher Scientific, Waltham, MA, USA). Correct constructs were propagated, plasmids were isolated (Plasmid Mini, A&A Biotechnology, Gdynia, Poland), and used for transformation. *Y. lipolytica* strain was transformed using a standard lithium acetate method [100]. Transformants were selected as instructed by [99], so the co-transformation reactions were inoculated into 9 mL of YPD-hygromycin-nourseothricin liquid medium and cultured at 28 °C for 48 h with shaking at 150 rpm. The following selection conditions were used: hygromycin B at 400 μg L^−1^, or nourseothricin (both from Sigma-Aldrich-Merck-Millipore, Darmstadt, Germany) at 250 μg L^−1^, supplemented to YPD medium (liquid or solidified). One mL of such cultures was then transferred into 9 mL of YPD-nourseothricin medium and incubated at 28 °C for 24 h with shaking at 150 rpm to allow plasmid curing (dropping-off JME4580). Finally, the culture was diluted and plated on YPD-nourseothricin agar. Clones appearing after 48 h of incubation at 28 °C were verified for correct integration of the deletion cassettes by PCR and sequencing. All the strains were deposited as 30% glycerol stocks at −80 °C for long-term storage.

### 4.3. Functional Studies—Direct Comparative Study of OE and KO Strains

#### 4.3.1. Cultivation Conditions

The yeast strains were revived from glycerol stocks and then routinely maintained at 28 °C in rich YPD (g L-1: yeast extract, 5 (BTL, Łódź, Poland); peptone, 10 (BTL); glucose, 20 (POCH, Gliwice, Poland); solidified with agar, 15 (BTL)) or in minimal YNB medium (g L-1: glucose, 10 (POCH); yeast nitrogen base, 1.7 (Sigma-Aldrich, St. Louis, MI, USA); ammonium sulfate, 5 (POCH); solidified with agar, 15 (BTL)). Two to six subclones were cultivated in biological duplicates according to a high-throughput cultivation protocol developed in our laboratory [23].

The cultivation medium and conditions were as follows [g L-1]: yeast nitrogen base without amino acids and ammonium sulfate, 5.1 (Sigma-Aldrich); ammonium sulfate and glutamic acid, both at 7.5 (POCH); glucose, 35 (POCH); buffered with 0.2 M maleic acid at pH 5.0 (POCH). Precultures were developed in media composed of a yeast nitrogen base, 5.1 (Sigma-Aldrich); ammonium sulfate, 15, (POCH); glucose, 25 (POCH); buffered with 0.2 M maleic acid at pH 5.0. All the media were filter-sterilized with 0.22-μm filters (Merck-Millipore, Darmstadt, Germany).

#### 4.3.2. Samples Analysis

Samples were withdrawn at 48 h and assessed for optical density (OD) and fluorescence (FL). Before reading the FL from the reporter protein (RedStar2), samples were diluted in 0.75% NaCl (POCH) to match a linear range of the methods. Absorbance was measured at 600 nm in transparent 96-well plates (Costar; Merck-Millipore, Darmstadt, Germany). FL was determined under ex/em wavelengths 550/595 nm in black opaque plates (Thermo Fisher Scientific). Both measurements were performed using a Tecan Spark automatic plate reader (Tecan Group Ltd., Mannedorf, Switzerland).

Fold change values were calculated by dividing the raw readouts for the TF-engineered strain by the result read for the control strain (overexpressing only in RedStar2 protein, with no TF modification).

### 4.4. Omics and Functional Data Acquisition

Transcriptomics data were extracted from the NCBI SRA database (PRJNA701856 and PRJNA869113). Data were filtered for gene identifiers of the TF-encoding genes based on [20]. For the non-significantly deregulated genes, a default value of 0 was set.

Phenotype OE screen data were extracted from the YaliFunTome database (https://sparrow.up.poznan.pl/tsdatabase/, accessed throughout June 2024) and accompanying publication [23]. The phenotype data were chosen from a variant of high oxygen availability, pH 5, temperature 28 °C, glucose as a carbon source, and the average of ammonium sulfate (AS) and casamino-acid hydrolysate (CH) as nitrogen sources.

### 4.5. Data Processing and Mathematical Analysis

The results for each TF gene are presented in the form of a heatmap. K-means clustering was performed on these data, with the number of clusters = 10 (determined by an ‘elbow method’, Supplementary Material—Figure S1), random state = 42, scaled with StandardScaler (sklearn package version 1.3.2). The genes are sorted according to clusters, to which they have been assigned. Clusters are separated with bold horizontal lines (Figure 1). All the analyses were performed in Visual Studio Code (version 1.92.2) in the Python programming language (version 3.11.9) with corresponding data-processing and machine-learning packages.

The correlation matrix for the TFs displaying a universal deregulation pattern in the transcriptomics datasets (presented in Figure S2) was calculated based on the TFs’ transcriptional profile in the five strains and on TF-co-OEing (each in two parameters: total FC rProt synthesis and normalized to biomass FC rProt synthesis). The statistical significance of each correlation was calculated with the Pearson r-coefficient at a *p*-value < 0.05.

A statistical overrepresentation test was run using Panther [67,68]. Analysis Type: PANTHER Overrepresentation Test (Released 20240226). Annotation Version and Release Date: GO Ontology Database DOI: 10.5281/zenodo.10536401 Released 2024-01-17. Analyzed List—YALI signatures of 139 TFs under study (Table 1). Reference List—The reference gene list for the test. *Y. lipolytica* (all genes in database, 6448), 138 user-defined IDs were mapped to the genome. Annotation Dataset—GO biological process complete—Complete GO biological process annotations, including both manually curated and electronic annotations. Electronic annotations were generated by computer algorithms based on sequence similarity. This is the test that was performed using Fisher’s exact test with FDR correction.

Statistical significance of the differences observed between OE and KO strains vs. the control strain (JMY2810) was assessed by the ANOVA analysis of variance and post hoc Tukey test at a significance level of *p* < 0.05 (Visual Studio Code, version 1.92.2).

## Figures and Tables

**Table 1:** *Y. lipolytica* TFs with putative or assigned function.

Yali Signature	Assigned Name	Putative/Known Function	Reference
*B12716*	*HAC1*	Transcriptional activator of genes involved in ER-based Unfolded Protein Response (UPR).	[16,19]
*E31757*	*BRG1*	Biofilm regulator 1.	[20]
*D14520*	*SKN7*	TF involved in the activation of osmotic and oxidative stress.	[22,40]
*D07744*	*YAP-like*	TF involved in pH-dependent dimorphic transition and maintaining redox balance.	[22,41]
*D20482*	*GZF1*	Inducer of the nitrogen catabolite repression (NCR) genes.	[23,42]
*E13948*	*HSF1*	Heat shock transcription factor.	[22,23]
*D05041*	*KLF1*	Krueppel-like factor 15, regulates the expression of genes for gluconeogenic and amino acid-degrading enzymes.	[23]
*A16841*	*AZF1*	Asparagine-rich Zinc Finger protein, regulates carbon metabolism in yeast and cell wall organization.	[20,23]
*F05896*	*DEP1*	Part of the Rpd3C(L) histone deacetylase complex (HDAC). Transcriptional modulator involved in regulation of structural phospholipid biosynthesis genes.	[20,23]
*C19151*	*CAT8*	CATabolite repression TF 8. Inducer of gluconeogesis, maintains energy homeostasis, presumed to regulate formate dehydrogenases expression.	[39]
*F21923*	*ADR1*	Alcohol Dehydrogenase II synthesis Regulator, inducer of genes involved in alternative carbon utilization upon glucose starvation.	[11,39]
*D20460*	*LAC9*	LACtose regulatory protein, controls induction of the lactose-galactose regulation.	[23]
*B14443*	*JMC2*	JmjC domain family histone demethylase, promotes global demethylation of H_3_K_4_.	[23,43]
*D02783*	*DAL81*	Positive regulator of genes in multiple nitrogen degradation pathways, involved in nitrogen catabolite activation of transcription from RNA polymerase II promoter.	[23]
*B19602*	*MGF2*	Mycelial growth factor 2. TF of known roles in the dimorphic transition.	[16,41]
*B21582*	*MHY1*	Mns2/Mns4-like protein, a key regulator of yeast-to hypha dimorphic transition but not stress resistance, regulates both alkaline-pH and glucose-induced filamentation.	[44,45,46]
*D01573*	*MGF1*	Mycelial growth factor. Potential driver of the transition between morphological phases.	[16]
*B05038*	*ZNC1*	Zinc finger transcriptional factor, regulates the yeast-to-hypha transition in the dimorphic yeast.	[47]
*F17886*	*GZF2*	GATA—binding zinc finger transcription factor 2, inducer of NCR, essential for growth on simple nitrogen sources.	[42]
*C22682*	*GZF3*	GATA-zinc finger transcription factor 3, repressor of NCR.	[20,42]
*E05555*	*GZF4*	GATA-zinc finger transcription factor 4. Putative: inducer of NCR.	[42]
*E16577*	*GZF5*	Non-genuine GATA-zinc finger transcription factor.	[20,42]
*E03410*	*ERT1-2*	Positive regulator of gluconeogenesis.	[20]
*E27742*	*GCN4*	General Control Non-derepressible 4 TF. Key transcriptional activator of amino acid biosynthesis genes.	[48]
*F17424*	*HAP1*	TF responsible for oxygen sensing and signaling.	[49]
*D04785*	*SFL1*	Repressor of filamentous growth and flocculation.	
*F01562*	*EUF1*	Transcription factor mediating expression of erythritol synthesis genes.	[11,50,51]
*C16863*	*SKO1*	*ATF/CREB* family transcription factor, repressor that mediates *HOG* pathway-dependent regulation of osmotic stress response, involved in protection from oxidative damage.	[40]
*C14784*	*YAS3*	Transcriptional repressor of *ALK* genes, de-repressed on alkanes.	[52]
*C13750*	*MSN4*	General stress response, regulates tolerance to acid-induced stress. Regulates genes involved in the antioxidant cellular response.	[53]
*B13640*	*RIM101*	pH-response transcription factor, regulator of alkaline-induced filamentation	[46]
*C12364*	*NRG1*	*NRG1* repressor of erythritol utilization genes, plays a minor role in repression of filamentation.	[50,54]
*D23749*	*ZAP1*	*ZAP1* involved in zinc ion homeostasis by zinc-responsive transcriptional regulation.	[20]
*E30789*		Putative^a^: transcriptional regulator of form adherence 5.	
*C07821*		Putative^a^: glucose transport transcription regulator *RGT1*-related.	
*C18645*	*ARO80*	Transcription activator required for the expression of genes involved in the catabolism of aromatic amino acids.	[55]
*B08206*	*CRF1*	Copper resistance protein transcriptional regulator.	[56]
*D14872*		Putative ^a^: transcriptional regulatory protein *STB4*	
*E18304*	*ERT1-1*	Transcription activator of gluconeogenesis.	[20]
*E07942*	*MIG1*	Controls genes involved in beta-oxidation, involved in carbon catabolite repression.	[57]
*A18469*	*HOY1*	Homeobox protein, a positive regulator of hyphae formation.	[20,58]
*B06853*	*PUT3*	*PUT3* proline utilization trans-activator.	
*B09713*	*PPR1*	*PPR1* pyrimidine pathway regulatory protein, de novo biosynthesis.	[54,59]
*D13904*	*LEU3*	Leucine-responsive transcription regulator, regulates genes involved in branched-chain amino acid biosynthesis and ammonia assimilation.	
*D23045*	*AHR1*	*AHR1* adhesion and hyphal regulator 1.	
*E10681*	*WAR1*	Weak Acid Resistance transcription factor.	
*E20449*	*YOX1*	Homeobox protein *YOX1*. Transcriptional repressor of ECB-dependent genes (early cell box) to the G1/M phase.	
*F03157*	*MET32*	*MET32* auxiliary transcriptional regulator of sulfonate and sulfur amino acid metabolism, methionine biosynthesis, and sulfate assimilation.	
*F05104*	*TFIIIA*	*PZF1* general transcription factor IIIA.	
*F09361*	*U4/U6.U5* *component*	U4/U6.U5 tri-snRNP, spliceosomal complex—may play a role in mRNA splicing.	
*F09493*	*SAP61*	Pre-mRNA-splicing factor sap61, involved in mRNA splicing, associates with cdc5 and the other cwf proteins as part of the spliceosome.	
*F16599*	*STB4*	Putative transcription factor *STB4*—Sin Three Binding protein, involved in the transcription of transmembrane transporters.	[60]
*F18788*	*RLM1*	May function as a TF downstream of *MPK1*, at least some RML1 target genes are involved in cell wall biosynthesis.	
*E31845*	*PRZ1*	Involved in the regulation of calcium ion homeostasis.	
*B15818*	*SterTF*	Sterol transcription factor, regulating sterol biogenesis.	[61]
*D05005*	*SEF1*	Zn2-Cys6 transcription factor; regulates iron uptake.	
*D12628*	*POR1*	Primary oleate regulator 1—transcriptional activator regulating genes involved in fatty acid utilization.	[62]
*C02387*	*YAS1*	Transcription factor essential for cytochrome p450 induction in response to alcanes, heteromeric Yas1p/Yas2p complex transcription factor.	[63]
*B08734*	*REI1*	Cytoplasmic pre-60S factor *REI1* involved in maturation of the ribosomal 60S subunit.	
*E01606*	*OAF3*	Oleate activated transcription factor 3, transcriptional inhibitor with a significantly increased number of target genes in response to oleate.	
*C06842*	*MCM1*	*MCM1* transcription factor involved in biofilm formation, cell adhesion, and hyphal growth.	
*A19778*	*MBP1*	MluI-box Binding Protein, involved in regulation of cell cycle progression from G1 to S phase.	[39,53]
*D15334*	*CPH2*	Transcription factor that positively controls filamentous growth.	
*D01463*	*CRZ1*	*CRZ1* transcription regulator involved in the regulation of calcium ion homeostasis.	
*D13068*	*BUD20*	*BUD20* bud site selection protein 20—positioning the proximal bud pole signal; protein required for ribosome assembly.	
*B13354*	*AP-4*	Transcription factor that activates viral and cellular genes.	
*F25861*	*RPN4*	*RPN4* transcription factor regulating proteasomal genes.	
*D24167*	*CBF1*	*CBF1* centromere binding factor 1, required for chromosome stability and chromosomal segregation.	
*C12639*	*SWI6*	*SWI6* part of a complex involved in cell-cycle-dependent transcription. *SWI4* and *SWI6* are required for formation of the cell-cycle box factor-DNA complex	
*E25960*	*SWI1*	*SWI/SNF* chromatin-remodeling complex subunit *SWI1*.	
*F13321*	*OAF1*	Oleate activated transcription factor 1, activates transcription of genes involved in fatty acid beta-oxidation.	
*F11487*	*STP1*	Nutrient- and stress-responsive activator of ribosome biogenesis genes.	
*B05478*	*STP3*	Possibly involved in pre-tRNA splicing and in uptake of branched-chain amino acids.	[59]
*E15510*		Putative ^a^: Homeobox protein *YOX1*.	
*C22990*		Putative ^a^: *ASG1* general activator of stress genes.	
*D04466*		Putative ^a^: Regulatory protein cys-3. Turns on the expression of structural genes which encode sulfur-catabolic enzymes.	
*D09647*		Putative ^a^: Arginine metabolism regulation protein II. With *ARG80*, *ARG82* and *MCM1*, coordinates the expression of arginine anabolic and catabolic genes in response to arginine.	
*D10681*		Putative ^a^: Adhesion and hyphal regulator 1.	
*D18678*		Putative ^a^: Respiration factor 2. Transcription factor that regulates expression genes required for glycerol-based growth and respiration.	
*E17721*		Putative ^a^: Phosphatidylinositol N-acetylglucosaminyltransferase subunit P.	
*E31669*		Putative ^a^: Metal-binding activator 1. Copper ion-sensing transcription factor, promotes filamentous and invasive growth.	
*F05126*		Putative ^a^: Phosphorus acquisition-controlling protein.	
*F17468*		Putative ^a^: Multidrug resistance regulator 1. Acts as the central regulator of the *MDR1* efflux pump.	
*E11693*		Putative ^a^: TF required for repression of genes during iron starvation. Represses iron-dependent and mitochondrial-localized activities including respiration, TCA cycle, amino acid metabolism, iron-sulfur-cluster and heme biosynthesis.	
*E10131*		Putative ^a^: Transcription elongation factor 1. Implicated in the maintenance of proper chromatin structure in actively transcribed regions.	
*D09757*		Putative ^a^: AP-1-like transcription factor.	
*F05346*		Putative ^a^: Binds a palindromic promoter element essential for induction of fungal cutinase gene.	
*C11858*		Putative ^a^: DNA-binding transcription factor Moc3.	
*B15312*		Putative ^a^: May act as a co-chaperone for *HSP70*.	
*C18667*		Putative ^a^: Transcriptional activator of the arabinanolytic system.	
*E16973*		Putative ^a^: Metallothionein expression activator.	
*F11979*		Putative ^a^: Retrograde regulation protein 1.	
*C19063*		Putative ^a^: Transcription factor that mediates stress and developmental response.	
*F18326, E05577, B08360,* *C20977, C13794*		Putative ^a^: sterol uptake control.	

^a^ assignment of genes’ names and genes’ functions was manually curated by cross-referencing several databases—anther, KEGG, UniProt, and NCBI and blasting (blastp) the sequences against the databases’ collections; The following TFs have no assigned function in the databases mentioned above: *A10637, A12925, B00660, B20944, C01375, C03564, C05995, C09009, C09482, C13178, C15202, C16390, D01353, D01419, D06952, E17215, E18161, E18656, E24277, E31383, F06072, F13695, F15037, D09757, B04510, F22649, D17988, F03135, B22176, F11011, E19965, F15543, E14971, E20251, F13761, E34925, E23518, B13200, E10087, F16852, D02475, D06193*. The references are given for functional studies in *Y. lipolytica*.

## Data Availability

The primary transcriptomics datasets used in this study are openly available in the NCBI SRA database at https://www.ncbi.nlm.nih.gov/sra/, under reference numbers PRJNA701856 and PRJNA869113. The primary functional screen datasets used in this study are openly available in the YaliFunTome database at https://sparrow.up.poznan.pl/tsdatabase/.

## Supplementary Materials

The following supporting information can be downloaded at: https://www.mdpi.com/article/10.3390/ijms25179450/s1, Figure S1. Graphic representation of running an “Elbow method” used for determination of the optimal number of clusters in k-means clustering of transcriptomic and phenotype data for TFs analyzed in this study. Figure S2. The correlation matrix demonstrates the similarity of the transcriptional profiles and the functional screen profiles between the analyzed samples for the TFs displaying a uniform deregulation pattern in the transcriptomic data. Table S1. Results of Statistical Overrepresentation Test.
