# Supplementary material for: An Interplay between Transcription Factors and Recombinant Protein Synthesis in Yarrowia lipolytica at Transcriptional and Functional Levels—The Global View
Source: Int J Mol Sci. 2024 Aug 30;25(17):9450. doi: 10.3390/ijms25179450 (PMC11395014; doi:10.3390/ijms25179450)

Supplementary material

Figure S1. Graphic representation of running an "Elbow method" used for determination of the optimal number of clusters in k-means clustering of transcriptomic and phenotype data for TFs analyzed in this study.

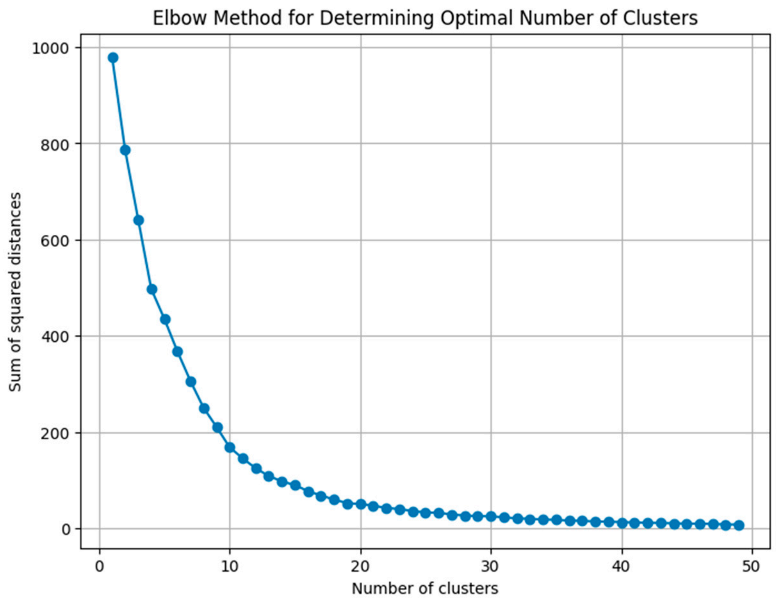

Figure S2. The correlation matrix demonstrating the similarity of the transcriptional profiles and the functional screen profiles between the analyzed samples for the **TFs displaying a uniform deregulation pattern** in the transcriptomic data (**Figure 4**). Yellow framed – statistically significant correlation between functional screen results and the omics data.

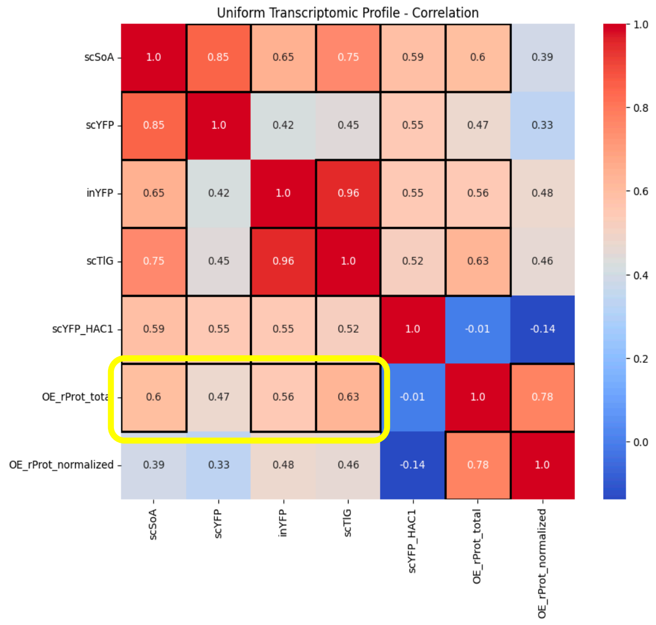

Supplement: Supplementary file 1 [file ijms-25-09450-s001.zip › ijms-3160738-supplementary.pdf]
